# Simultaneous RNAi Knockdown of Three FMRFamide-Like Peptide Genes, *Mi-flp1, Mi-flp12*, and *Mi-flp18* Provides Resistance to Root-Knot Nematode, *Meloidogyne incognita*

**DOI:** 10.3389/fmicb.2020.573916

**Published:** 2020-10-23

**Authors:** Prakash Banakar, Alkesh Hada, Pradeep K. Papolu, Uma Rao

**Affiliations:** ^1^Division of Nematology, ICAR-Indian Agricultural Research Institute, New Delhi, India; ^2^Department of Nematology and Centre for Bio-Nanotechnology, Chaudhary Charan Singh Haryana Agricultural University, Hisar, India

**Keywords:** root-knot nematodes, FMRFamide-like peptides, neuromotor functions, double-stranded RNA, combinatorial RNAi, transgenic plants

## Abstract

Root-knot nematode, *Meloidogyne incognita*, is a devastating sedentary endoparasite that causes considerable damage to agricultural crops worldwide. Modern approaches targeting the physiological processes have confirmed the potential of FMRFamide like peptide (FLPs) family of neuromotor genes for nematode management. Here, we assessed the knock down effect of *Mi-flp1, Mi-flp12*, and *Mi-flp18* of *M. incognita* and their combinatorial fusion cassette on infection and reproduction. Comparative developmental profiling revealed higher expression of all three FLPs in the infective 2nd stage juveniles (J2s). Further, *Mi-flp1* expression in J2s could be localized in the ventral pharyngeal nerves near to metacarpal bulb of the central nervous system. *In vitro* RNAi silencing of three FLPs and their fusion cassette in *M. incognita* J2s showed that combinatorial silencing is the most effective and affected nematode host recognition followed by reduced penetration ability and subsequent infection into tomato and adzuki bean roots. Northern blot analysis of J2s soaked in fusion dsRNA revealed the presence of siRNA of all three target FLPs establishing successful processing of fusion gene dsRNA in the J2s. Further, evaluation of the fusion gene cassette is done through host-delivered RNAi in tobacco. Transgenic plants with fusion gene RNA-expressing vector were generated in which transgene integration was confirmed by PCR, qRT-PCR, and Southern blot analysis. Transcript accumulation of three FLPs constituting the fusion gene was reduced in the *M. incognita* females collected from the transgenic plants that provided additional evidence for successful gene silencing. Evaluation of positive T_1_ transgenic lines against *M. incognita* brought down the disease burden as indicated by various disease parameters that ultimately reduced the nematode multiplication factor (MF) by 85% compared to the wild-type plants. The study establishes the possibility of simultaneous silencing of more than one FLPs gene for effective management of *M. incognita*.

## Introduction

Plant-parasitic nematodes (PPNs) have proven to be one of the most challenging to control and stubborn pests causing an estimated yield loss amounting to $US 173 billion globally (Elling, 2013). *Meloidogyne incognita*, is the most widespread and damaging root-knot nematode (RKN) species in the world causing serious consequence for food security and economy of the farming community (Trudgill and Blok, 2001; Jones et al., 2013). *Meloidogyne* species are soil borne root feeders that cause severe root galling, stunted plant growth due to impaired nutrient absorption and inclination of roots to other pathogen invasion (Jones et al., 2013).

Considering the management strategies available to mitigate the RKNs, it is very challenging to select any single promising tool which is effective, eco-friendly and harmless to the non-target organisms (Bridge et al., 2005; De Waele et al., 2013). Genetic improvement through traditional breeding program faces various challenges due to non-availability of appropriate resistant source for a given crop. Management of PPNs currently relies greatly on resistant plants developed through conventional breeding methods. Further, evolution of races and pathotypes within the nematode population restricted the effectiveness of existing resistant genotypes, and selection pressure often leads to resistance-breaking biotypes of the pests. Chemical nematicides have widely been used to manage these PPNs. The recent introduction of nematicidal chemicals, *viz.*, fluensulfone, fluazaindolizine, tioxazafen, and fluopyrum although have been claimed to be environmentally friendly and well-targeted against RKNs (Kearn et al., 2014; Slomczynska et al., 2014; Faske and Hurd, 2015; Lahm et al., 2017), the adverse effects of chemical pesticides cannot be overruled (Starr et al., 2002). Therefore, there has been a continuing demand for the progression of target-specific, environmentally sound and biodegradable pest management approaches and genetic engineering based techniques have emerged as a precious alternative and corresponding approach in this regard (Fairbairn et al., 2007; Danchin et al., 2013; Roderick et al., 2018; Chaudhary et al., 2019; Hada et al., 2020).

Targeting genes critical for the survival of nematodes provide environmentally sound strategy. Availability of *M. incognita* genome and several ESTs offers a repository for identification of candidate genes responsible for nematodes parasitism (McCarter et al., 2005; Abad et al., 2008). The knowledge of nematode genes responsible for successful infection and parasitism inside a host can give clue for arresting some vital physiological processes required for completing its life cycle.

The nematodes neuropeptides play many vital roles in modulating motor and sensory system and, regulate various physiological and behavioral processes including host recognition, infection, alimentation and reproduction. Neuropeptide signaling system has been identified as potential drug target due to its established effect in host recognition and infection inside the host (Maule et al., 2002; Kimber et al., 2007; Papolu et al., 2013).

FMRFamide-like peptides (FLPs) establish the most diverse and largest group of neuropeptides, leading to behavioral changes of nematodes by binding through G-protein coupled receptors (GPCRs) or peptide-gated ion channels (Husson et al., 2007; Atkinson L. E. et al., 2013; Rao et al., 2013). These FLPs transcripts are synthesized from *flp* genes on large propeptides, containing individual peptides in single or multiple copy number flanked by the cleavage sites having mono (R/K) or dibasic (KK/KR/RK/RR) amino acids. The propeptide molecules originated from the N-terminal signal sequences of peptide leads to synaptic drop of the mature peptide by secretory pathway (McVeigh et al., 2006). Fundamental physiological roles of a few FLPs are identified in *Caenorhabditis elegans* and *Ascaris suum* in their biology. However, most of the biological evidences on FLPs are lacking in PPNs, and *M. incognita* most likely due to their obligate nature which confines the use of conventional physiological procedures. Nineteen FLPs are reported from *M. incognita* out of which six have been established to have transcriptional confirmation *viz., Mi-flp1*, *Mi-flp7, Mi-flp12, Mi-flp14*, *Mi-flp16*, and *Mi-flp18* (Magrane and UniProt Consortium, 2011). Multiple Sequence alignment of these known FLPs in RKNs exhibited less sequence similarity among them despite they share a typical RF-amide sequence at C-terminus. Their uniqueness could consequently be advantageous at generating sequence specific knockdown module by dsRNA approach to avoid any off target effects.

Increasing evidence for the development of RNAi based transgenics suggests that expression of *M. incognita* specific dsRNA molecule in the host plant may provide alternative management strategy. The simplicity and targeted knock down effect associated with RNAi has immense value for deciphering the gene function in many organisms including the obligate PPNs (Urwin et al., 2002; Rosso et al., 2005; Kimber et al., 2007; Shingles et al., 2007; Dalzell et al., 2009; Ibrahim et al., 2011; Niu et al., 2012; Chaudhary et al., 2019). Rosso et al. (2005) explored the RNAi for the first time in *M. incognita* J2s for the silencing of two genes, *Mi-crt* and *Mi-pg1* which are expressed in the sub-ventral esophageal glands of the nematodes and probably involved in early parasitism.

Investigation on the FLPs genes function through RNAi has shown reduced migration, penetration and aberrant behavioral phenotypes in *M. incognita, Meloidogyne graminicola*, and *Globodera pallida* (Urwin et al., 2002; Kimber et al., 2007; Dalzell et al., 2010; Atkinson L. E. et al., 2013; Papolu et al., 2013; Dong et al., 2014; Banakar et al., 2015; Kumari et al., 2017). Further, effectiveness of *in vitro* RNAi of FLP genes (*Gp-flp1, Gp-flp12*, and *Gp-flp18*) in *G. pallida* (Kimber et al., 2007) and host-delivered RNAi of *Mi-flp14, Mi-flp18* in *M. incognita* (Papolu et al., 2013) indicate that it would be intriguing to investigate the synergistic or additive impacts, if any, of concomitant knockdown of more than one *flp* gene. Multiple genes silencing concurrently has been successfully employed in *C. elegans* (Tischler et al., 2006; Gouda et al., 2010; Min et al., 2010). Tischler et al. (2006) simultaneously silenced four genes *viz., lin-31, sma-4, unc-22*, and *lon-2* having different biological functions through RNAi by feeding modified bacteria to *C. elegans* that produced comparatively weaker phenotypes compared to the double genes. Gene pyramiding in plants has been proven to be a better approach for resistance against many pathogens which cause substantial yield losses (Urwin et al., 1998; Zhao et al., 2003; Abdeen et al., 2005; Turra et al., 2009; Roderick et al., 2012; de Souza Júnior et al., 2013; Walawage et al., 2013; Narusaka et al., 2014; Quilis et al., 2014; Tripathi et al., 2014; Chan et al., 2015). Targeting multiple genes has also been utilized for the development of nematode resistant transgenic plants. Urwin et al. (1998) developed transgenic Arabidopsis plants with a stacked gene construct, having a CpTI and Oc-I_D86 genes. The transgenic plants exhibited superior resistance to *Heterodera schachtii.* Similarly, Chan et al. (2015) delivered a dual gene construct, which included CeCPI and a PjCHI-1 genes in transgenic tomato plants with synthetic promoter, pMSPOA, having NOS-like and SP8a elements. These transgenic tomato plants having dual genes significantly reduced the RKN infection and reproduction compared with the plants transformed with a single gene.

In view of the above, we have selected three *M. incognita* FLP coding genes, *Mi-flp1, Mi-flp12*, and *Mi-flp18* and their fusion sequence prepared synthetically for evaluating RNAi silencing effect on host finding, invasion and reproduction of *M. incognita*. Further, functional validation through host-delivered RNAi of FLPs-fusion gene in *Nicotiana tabacum* L. using *Agrobacterium tumefaciens-*mediated transformation and evaluation of transgenic plants against *M. incognita* has been undertaken. In addition, site of expression of a gene helps in better understanding of its function and hence expression site of *Mi-flp1* which is not known is deciphered in the present study. Thus, the present investigation strengthens our knowledge on the effect of *M. incognita Mi-flp1, Mi-flp12, Mi-flp18* and their fusion gene on nematode reproduction and plant parasitism potential when applied through host-delivered RNAi.

## Materials and Methods

### Nematode Culture

A pure population of *M. incognita* was maintained on eggplant (*Solanum melongena* cv. Pusa Purple Long) in the greenhouse. Further, Adzuki bean plants growing in CYG growth pouches^1^ infected with *M. incognita* were used for collecting the different developmental stages [eggs, pre parasitic juveniles (J2s), 3rd and 4th stage juveniles (J3s/J4s), and young adult females] of the nematode (Atkinson and Harris, 1989; Tyagi et al., 2013). Additionally, fresh egg masses from the infected plants were also collected for hatching the pre-parasitic J2s required for further experiments.

### Characterization of FLPs Transcripts in *M. incognita* J2s

Freshly hatched J2s of *M. incognita* were collected and total RNA was isolated using NucleoSpin RNA extraction kit (Macherey-Nagel, Germany). RNA quality and quantity was analyzed using NanoDrop-2000 (Thermo Fisher Scientific, United States) and about 500 ng of total RNA was reverse transcribed to complementary DNA (cDNA) with cDNA synthesis Kit (Superscript VILO, Invitrogen, United States).

To design the primers for FLPs, sequences of *Mi-flp1* (KC517344.1), *Mi-flp12* (AY804187.1), and *Mi-flp18* (AY729022.1) of *M. incognita* available in GenBank were used and aligned in the ClustalX version 1.81 (Thompson et al., 1997). Conserved nucleotide sequences were used and primers were designed using IDT OligoAnalyzer, and primer sequences are given in Supplementary Table S1. Selected genes were PCR amplified from cDNA and cloned into pGEM-T easy vector (Promega, United States) and confirmed the target gene inserts by sequencing. Selected sequences of *Mi-flp1, Mi-flp12*, and *Mi-flp18* were further used for designing the fusion gene cassette by placing the three sequences continuously and synthesized artificially by Biomatik Custom Gene Synthesis (Biomatik Technologies, United States) and sub-cloned into pGEM-T easy vector (Promega, United States).

### Comparative Expression of FLPs in Different Developmental Stages of *M. incognita*

Different developmental stages of *M. incognita* (eggs, pre parasitic J2s, J3s/J4s, and young adult females) were collected and used for RNA extraction and cDNA preparation as elaborated above. Quantitative Real-time PCR (qRT-PCR) primers (Supplementary Table S1) were also designed using IDT OligoAnalyzer. qRT-PCR was carried out with SYBR Green PCR Master Mix (Eurogentec, Liège, Belgium) in realplex2 thermal cycler (Eppendorf, Germany). The conditions and melt curve program for qRT-PCR reaction was similar as previously depicted by Papolu et al. (2013). Relative fold-change in target gene expression was analyzed using comparative Ct method (Livak and Schmittgen, 2001) and log2- transformed. Egg stage gene expression was used as base level expression for comparing with other developmental stages. *M. incognita 18S rRNA* (*Accessions-HE667742*) was taken as a reference gene for the gene expression normalization. Three biological and three technical replicates were used for each of the samples during qRT-PCR analysis.

### *In situ* Hybridization for Localization of Site of Expression of *Mi-flp1*

The plasmids harboring the target cDNA sequence of *flp1* was used as a template to amplify the gene. Subsequently, PCR product was used for preparing the DIG- labeled sense and antisense DNA probes, separately (Roche, Switzerland). J2s fixation, permeabilization, hybridization of probes and signal detection and staining procedures were followed as described by Banakar et al. (2015). Processed nematodes were placed on microscope slides, covered with 0.28 mm glass coverslips and sealed with nail varnish. The observations were recorded in the Zeiss Imager M2m compound microscope. Images were recorded with Axion camera MRc5 through Axion vision Rel. 4.8 software (Zeiss Imager M2m, Carl Zeiss, Germany).

### DsRNA Synthesis and *in vitro* Silencing of FLPs Genes in *M. incognita* J2s

Selected FLP genes were amplified from the pGEM-T clones using M13 universal primers. Double-stranded RNAs (dsRNAs) were synthesized for each of the target genes as described earlier by Shivakumara et al. (2016), with minor modifications as 2 μl of labeled dUTP with Alexa Flore 488 (10 mM) was used. Additionally, dsRNA of GFP (green fluorescent protein, GenBank: HF675000) was prepared to be used as negative control. Synthesis of dsRNA was confirmed by resolving on 1% (w/v) agarose gel and visualized in gel documentation system (AlphaImager^®^ analyzer, United States).

About 2000 Freshly hatched J2s were soaked in 100 μl soaking solution having 50 μl of 50 mM octopamine (DL-octopamine hydrochloride, Sigma, United Kingdom), 2 μl labeled dsRNA (1 μg/μl) and 48 μl nuclease free water (Urwin et al., 2002) for 18 h in dark on a rotator (10 rpm) at room temperature. dsRNA soaking was done for all the target FLPs singly and combinatorial fusion gene in triplicates. J2s incubated separately in dsRNA of GFP and water were used as negative controls. Fluorescence was measured using EnSpire^®^ Multimode Plate Reader (PerkinElmer, United States) with default settings and excitation wavelength 488 λ and emission wavelength was 520 λ. The dsRNA soaked worms were placed in the 96 well black OptiPlate^TM^-96 F (PerkinElmer, United States). About 100 nematodes were used in each well with 12 replications for both control and dsRNA soaked worms. dsRNA uptake was also confirmed by microscopic observation using a ZEISS SteREO Discovery V20 microscope.

Juveniles soaked overnight in dsRNA solutions as aforesaid for different FLPs genes were washed with nuclease free water and frozen immediately in liquid nitrogen. Total RNA was isolated, quantified, and reverse transcribed. qRT-PCR has been carried out to quantify the transcript accumulation of all the target genes so as to confirm target-specific knockdown as described above. Three biological and three technical replicates were kept for each of the samples and gene expression was normalized using *18S rRNA*. Relative fold-change in target gene expression was analyzed using comparative Ct method (Livak and Schmittgen, 2001) and log2- transformed. Statistical analysis was carried out to assess the significance difference between treatments and water control worms.

### Northern Blot Analysis of *M. incognita* for siRNA Detection

Northern hybridization of the fusion dsRNA soaked J2s was done to confirm the processing of the dsRNA into siRNAs using a standard protocol (Sambrook et al., 1989). Nematodes were incubated in the fusion cassette dsRNA solution as mentioned above. Total small RNA was isolated from 100 mg of nematodes tissue using mirVana^TM^ miRNA Isolation Kit (Thermo Fisher Scientific, United States) and resolved on 30% denaturing PAGE (Polyacrylamide Gel Electrophoresis) gel and transferred onto a nitrocellulose membrane (Bio-Rad, United States) using iBlot^®^ gel transfer device (Invitrogen, United States). Probes of *Mi-flp1, Mi-flp12*, and *Mi-flp18* were separately prepared, hybridized, and detected as described earlier by Papolu et al. (2013).

### Effect of Gene Silencing on Nematode Attraction and Penetration in Pluronic F-127

To assess the knockdown effect of candidate genes on *M. incognita* infectivity, we evaluated penetration/invasion of dsRNA-soaked J2s in tomato roots on a soil less pluronic PF-127 medium (Wang et al., 2009; Chaudhary et al., 2019). Tomato seedlings were placed on pluronic gel medium in Petri plate and inoculated with dsRNA soaked J2s at a distance of 1.5 cm from the root tip. Sixteen replications were taken for each treatment. There were six treatments *viz., Mi-flp1* dsRNA, *Mi-flp12* dsRNA, *Mi-flp18* dsRNA, fusion cassette dsRNA, GFP dsRNA, and water control. Number of J2s that moved toward the root tips and present in the vicinity up to 1 mm diameter at different time intervals *viz*., 4, 8, 24, and 72 h were counted. Additionally, number of J2s penetrated at 24 and 72 h after inoculation was observed after roots staining with acid fuchsin (Byrd et al., 1983) and documented.

### Effect of Gene Silencing on Nematode Host Recognition and Penetration Assay in Soil

Tomato plants were grown in 18 cells seedling trays (JPP54, Jain Plasto Pack, India) for 25 days under greenhouse conditions. dsRNA feeding and washing steps were followed as mentioned above. For all the treatments and controls, 200 J2s were inoculated per plant (2 nematodes / g of soil) and provided 72 h for nematode penetration and were harvested. Roots were washed to remove the adhering soil and stained with acid fuchsin (Byrd et al., 1983) to calculate the number of invaded J2s in roots. There were six treatments as listed above and ten replications for each treatment.

### Functional Validation of FLPs on Nematode Infection, Development and Reproduction Through *in vitro* RNAi

Silencing effect of *Mi-flp1, Mi-flp12, Mi-flp18* and their synthetic fusion gene on *M. incognita* development and reproduction was carried out by inoculating dsRNA-soaked nematodes on 7-days old adzuki bean (*Vigna angularis* var. *angularis*) roots in CYG seed growth pouches (Banakar et al., 2015 and Shivakumara et al., 2016). Six root tips of each plant were inoculated with about 15–20 J2s, and incubated in a growth chamber at 27°C with 70% humidity and a light intensity of 300 lux (Jeio Tech Company Ltd., South Korea). Plants were supplemented with nutrient solution (Hoagland solution) after 7 days of inoculation at 3 days interval until harvest. At 35 DPI, plants were harvested, and disease burden per plant was scored using total number of galls, total number of endoparasites/females, number of egg masses, and number of eggs per egg mass, which were taken to calculate the multiplication factor (Papolu et al., 2013). Nematodes treated with GFP dsRNA and water were taken as controls. The experiments have three biological and ten technical replicates.

### Preparation of hpRNA Construct of Fusion Gene Cassette and Tobacco Transformation

On the basis of *in-vitro* RNAi outcome, the fusion gene cassette of FLPs containing *Mi-flp1, Mi-flp12*, and *Mi-flp18* was used for preparing hpRNA vector to examine host induced gene silencing effects. RNAi Gateway vector pB7GWIWG2(II) was procured from VIB-UGent Center for Plant Systems Biology, Ghent University, Ghent, Belgium. The commercially synthesized fusion gene cassette was PCR amplified from corresponding recombinant pGEMT clones and sub-cloned into the pDONR 221 (an entry vector). Subsequently, cloned into the destination vector “pB7GWIWG2(II)” using LR recombination in sense and antisense orientation intervening with an intron by GATEWAY recombination based cloning kit (Invitrogen, United States). The recombinant vector containing the hpRNA construct was transformed into *E. coli* (DH5α) cells. The positive clones were thereafter mobilized into *A. tumefaciens* strain EHA105, and confirmed the correct orientation of target gene by PCR using target gene specific primers, CaMV 35S promoter and attB2, CaMV 35S terminator and attB2, *bar* gene specific primers (Supplementary Table S1). Further, *A. tumefaciens* having fusion gene cassette was used for tobacco (*N. tabacum* L. cv. Petit Havana) transformation for functional validation as illustrated earlier by Papolu et al. (2013) and Shivakumara et al. (2019) with minor modifications.

### Molecular Characterization and Bioefficacy Analysis of Transgenic Plants

Putative transgenic plants expressing hairpin fusion gene cassette were subjected to molecular analysis to confirm the presence of transgene. For this, genomic DNA was isolated from young leaf tissues of various putative transgenic events and wild-type (WT) plants following NucleoSpin Plant II DNA kit (Macherey-Nagel, Germany). Primary confirmation of the transgene was done by performing PCR using different sets of primers (Supplementary Table S1). The amplified product was resolved on 1.0% (w/v) agarose gel.

To confirm the T-DNA integration and copy number, genomic DNA (∼12 μg) from the PCR-positive T_1_ plants was digested with *SacI* (20U/μl) restriction enzyme (New England Biolabs, United Kingdom) by incubating at 37°C for 16 h. Digested DNA samples were separated on 0.8% agarose gel, transferred onto a nitrocellulose membrane (Bio-Rad, United States). Fusion gene specific probe was synthesized and labeled with digoxigenin (DIG) probe labeling kit (Roche, Switzerland). Hybridization, washing, detection and blot development was done as earlier illustrated by Papolu et al. (2013).

The confirmed transgenic T_1_ lines were further subjected to qRT-PCR to interpret the FLPs transcripts abundance. Total RNA was isolated from the T_1_ plants leaves using NucleoSpin plant II RNA kit (Macherey-Nagel, Germany), about 500 ng of total RNA was converted to cDNA using Superscript VILO cDNA synthesis kit (Invitrogen, United States), and qRT-PCR was performed with the target genes specific primers in the realplex2 thermal cycler (Eppendorf, Germany). *N. tabacum 18S rRNA* (Accession: HQ384692.1) was used for gene expression normalization. Three biological and three technical replicates were taken for the study and data were analyzed following Livak and Schmittgen (2001).

Transgenic lines (T_1_) harboring hpRNA construct of fusion gene were subjected to nematode parasitism assays. 30-days old plants were grown in 4-inch diameter pots containing 200 g of soil and sand (5:1), each plant inoculated @ 2 *M. incognita* J2s per g of soil and maintained in a growth chamber at 28°C, 70% relative humidity, 14:10 h light:dark. Plants were harvested at 35 DPI, and the nematode parasitic success was documented as interprets earlier by Papolu et al. (2013).

Additionally, about 20 mature females were dissected out from the transgenics and WT plants, total RNA was extracted and reverse-transcribed (300 ng RNA) as mentioned above. Further transcripts accumulation was quantified using qRT-PCR with *Mi-flp1, Mi-flp12*, and *Mi-flp18* and *M. incognita 18S rRNA* was used as reference gene. Three biological and three technical replicates were considered for the study. Fold change in expression was analyzed using 2^–Δ^ ^Δ^ ^*CT*^ (Livak and Schmittgen, 2001).

### Statistical Analysis

The experimental data were analyzed using one-way analysis of variance (ANOVA) with completely randomized design (CRD), and significance was decisive at *P* = 0.05 and *P* = 0.01. The mean values of treatments were subjected to Duncan Multiple Range Significant Difference Test (DMRT).

## Results

### Functional Validation of FLP Genes Using RNAi

#### Cloning and Differential Expression of FLP Genes of *M. incognita*

**FIGURE 1:**
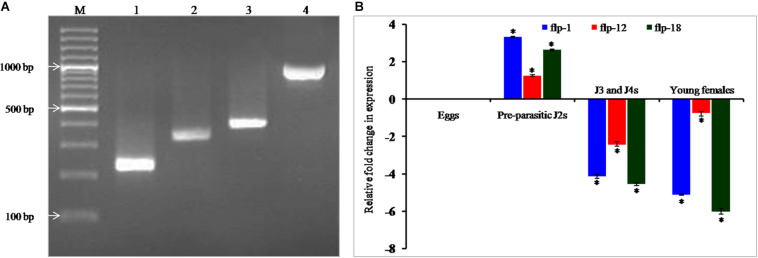
**(A)** PCR amplification of FLPs of *M. incognita.* Lanes – **1**: *Mi-flp1*, **2**: *Mi-flp12*, **3**: *Mi-flp18*, **4**: FLPs-fusion cassette, **M:** 100 bp marker, **(B)** Relative fold-change in the target genes expression in different developmental stages of *M. incognita*. Each bar represents the mean ± SE of *n* = 3, and asterisks indicate significant difference at *P* < 0.05.

Polymerase chain reaction (PCR) amplification, cloning and sequencing of 232, 349, and 407 bp sequences of *Mi-flp1, Mi-flp12*, and *Mi-flp18* amplified from the cDNA revealed 100% similarity with the previously reported sequences in NCBI database (Figure 1A). Differential expression of FLP genes in different developmental stages of *M. incognita* was performed by qRT-PCR. Based on expression of all three FLPs in eggs as reference, we observed that all three FLPs were significantly (*P* < 0.05) upregulated in the pre-parasitic J2s in relation to consistent down-regulation in J3/J4 and adult females (Figure 1B). Reference gene expression was constant in the different developmental stages. The expression stability was confirmed in three biological and three technical replications.

### *In situ* Hybridization of *Mi-flp1* in *M. incognita* J2s

**FIGURE 2:**
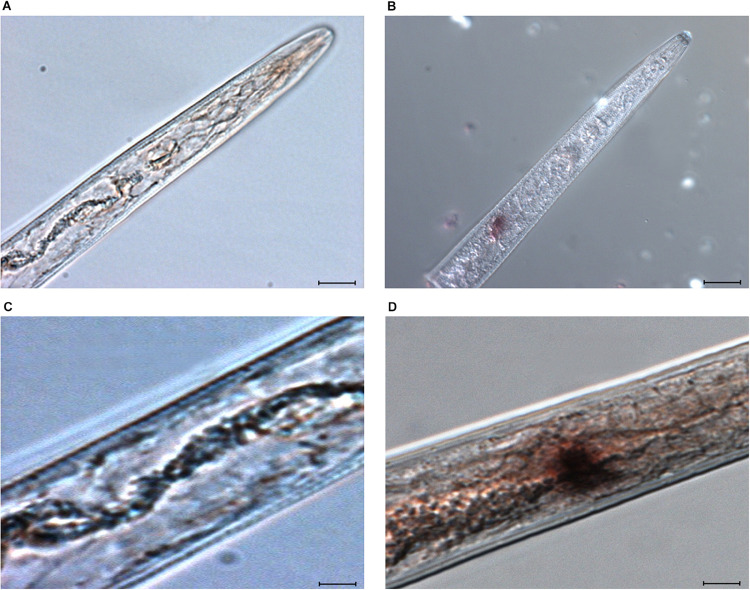
*In situ* hybridisation of DIG-labeled cDNA of *Mi-flp1* probe in the anterior of *M. incognita* J2s. **(A,C)** No hybridization signal in the control juveniles with DIG labeled sense probes, **(B,D)** Intense staining was observed in the nematode nerve ring region, revealing the *Mi-flp1* gene expression in ventral pharyngeal nerves near to metacarpal bulb of the central nervous system. *Mi-flp1* expression site is indicated by brown/black color due to the enzymatic breakdown of a chromogenic substrate by alkaline phosphatase conjugated to anti-DIG antibody. Scale bar = 10 and 20 μm.

The localization of *Mi-flp1* expression was performed by *in situ hybridization* in the *M. incognita* J2s. A 232 bp probe generated for *Mi-flp1* hybridized to the ventral pharyngeal nerves of the metacorporeal bulb in the nervous system. Intense staining was observed in the ventral pharyngeal nerves of the metacorporeal bulb (Figure 2B). However, precise identification of neurons was difficult in this tightly packed region of the nervous system. So, the neural map of *C. elegans* was used for interpreting the site of expression of the *Mi-flp1* in *M. incognita* J2s (White et al., 1986). Accordingly, *Mi-flp1* gene expression appeared to be localized in the AVK, AVA, AVE, RIG, RMG, AIY, and AIA pharyngeal MN neurons of *M. incognita* J2s (Figure 2D). There was absence of a hybridization signal in the control juveniles with DIG labeled sense probes Figures 2A,C).

### Quantification of mRNA Levels in dsRNA Treated *M. incognita* Using qRT-PCR, Northern Blot Analysis for siRNA Detection and Quantification of dsRNA Uptake

**FIGURE 3:**
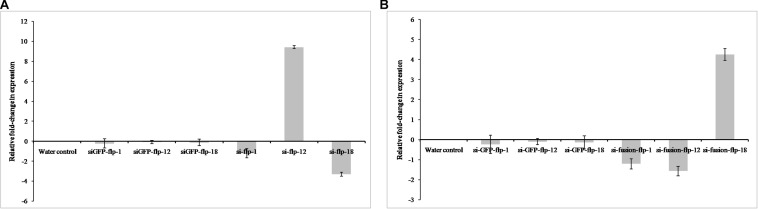
Quantification of target gene transcripts in dsRNA treated J2s of *M. incognita*. The relative expression levels of target mRNA were quantified by qRT-PCR in *M. incognita* silenced for **(A)** *Mi-flp1, Mi-flp12*, and *Mi-flp18*, **(B)** FLPs-fusion gene, compared to non-native GFP control and water control. *18s rRNA* was used as a reference gene and fold-change was calculated using 2^– Δ^ ^Δ^ ^*CT*^ method. Each bar represents the mean ± SE of *n* = 3 (*P* < 0.05).

The comparative expression levels of target mRNAs were quantified by qRT-PCR in nematodes silenced for *Mi-flp1, Mi-flp12, Mi-flp18*, and fusion gene compared to water control nematodes. Silencing of *Mi-flp1* and *Mi-flp18* singly led to down-regulation (*P* < 0.05) of the target genes compared to water control (Figure 3A). However, silencing of *Mi-flp12* singly resulted in significant (*P* < 0.05) up-regulation of the target gene over control. Silencing of the fusion gene having all three FLP genes (*Mi-flp1, Mi-flp12*, and *Mi-flp18*) resulted in significant (*P* < 0.05) down-regulation of *Mi-flp1* and *Mi-flp12* transcripts and up-regulation of the *Mi-flp18* (Figure 3B). Here, target gene specific knockdown could be confirmed with suitable controls and at first, none of the target genes was silenced in the nematodes incubated in GFP dsRNA. Reference gene expression was equal in both the dsRNA treated and water control worms. The expression stability was confirmed in three biological and three technical replications.

**FIGURE 4:**
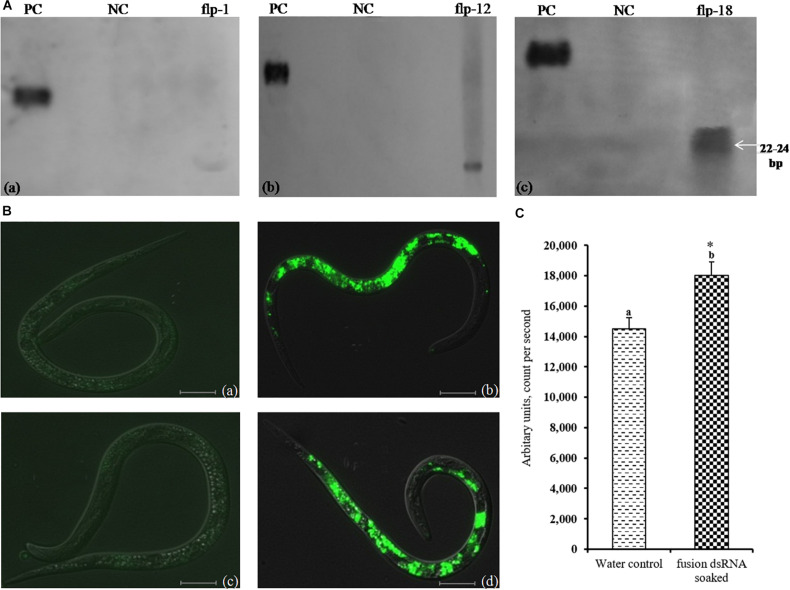
**(A)** Northern blot analysis for detection of siRNA in *M. incognita* J2s incubated in FLPs-fusion dsRNA. **(a)** *Mi-flp1*, **(b)** *Mi-flp12*, **(c)** *Mi-flp18*. PC – positive control (gene specific labeled probe: 25 pg); NC – negative control (total RNA from control healthy worms), **(B)** dsRNA uptake confirmation of *M. incognita* J2s incubated in FLPs-fusion dsRNA labeled with Alexa Flore 488 dUTPs using florescent microscope, **(a,c)** J2s without dsRNA, **(b,d)** dsRNA ingested J2s, fluorescence was localized in the stylet, esophageal gland cells, and intestine. Scale bar = 10 and 20 μm. **(C)** Difference in the fluorescence count between the water control and FLPs-fusion dsRNA soaked worms. Maximum fluorescence was quantified in 5000 flashes per minute using EnSpire^®^ Multimode Plate Reader (*t* = 2.306, *p* > 0000001). ^a,b,*^Represents the values are statistical significant (*t* = 2.306, *p* > 0000001).

As a key segment of successful RNAi, production of siRNAs of all three FLP genes was demonstrated by northern blot analysis in *M. incognita* J2s incubated in fusion dsRNA. The presence of siRNAs of all the target genes was abundant in fusion dsRNA soaked nematodes (Figure 4A). However, no expression was detected in water control nematodes.

Nematodes soaked in Alexa Flore 488 labeled florescent dsRNA showed fluorescence after 18 h of incubation and fluorescence was localized in the stylet, esophageal gland cells, and intestine (Figure 4B). Maximum fluorescence was quantified in 5000 flashes per minute in fluorescence multimode plate reader. The data were subjected to the Student’s *t*-test, and there was a significant difference between the water control and dsRNA soaked worms (Figure 4C) (*t* = 2.306, *p* > 0000001).

### Effect of *in vitro* RNAi on Attraction and Penetration of *M. incognita* Toward Tomato Roots on Pluronic PF-127 and in Soil

**FIGURE 5:**
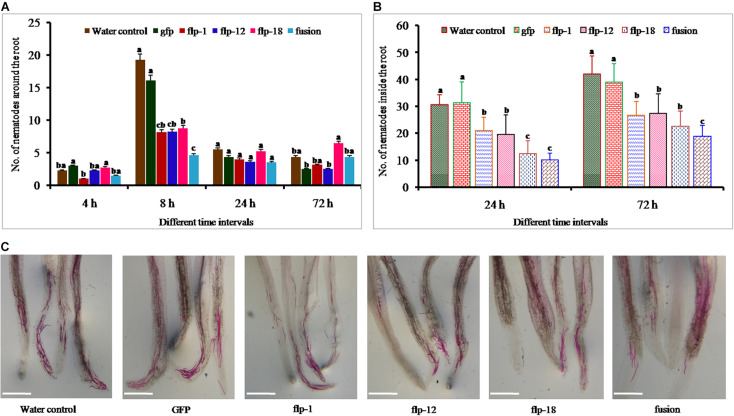
**(A)** Penetration ability of *Mi-flp1, Mi-flp12, Mi-flp18*, and FLPs-fusion dsRNA-soaked *M. incognita* J2s in tomato roots on PF-127 medium at 4, 8, 24, and 72 h. Each bar represents the mean standard error (*n* = 16); bars with letters stand for a significant difference at *P* > 0.05, **(B)** Number of nematodes inside tomato roots at 24 and 72 h of FLPs and GFP treated J2s and control worms in water, **(C)** Stained worms in infected tomato roots at 72 h post inoculation. Worms treated with non-native dsRNA (GFP) and worms in water were used as controls. Scale bar = 500 μm. ^a,b,c^Represents the values are statistical significant (*P* = 0.001).

Freshly hatched J2s of *M. incognita* were soaked in respective dsRNAs of the target genes for 18 h in the presence of 50 mM octopamine. In general, target genes silencing reduced *M. incognita* attraction to tomato roots compared to water control nematodes, and the effect was noticeable promptly after 4 h. Least number of nematodes were observed in fusion dsRNA soaked nematodes followed by *Mi-flp1* (*P* = 0.05), and a significant difference was observed between the fusion gene and single gene silenced nematodes at 8 h (Figure 5A). Silencing of each of the target genes decreased the *M. incognita* penetration/invasion at 24 and 72 h compared to the water control and GFP treatments (Figure 5B). However, simultaneous silencing of all the three FLPs through fusion gene provided the highest reduction in penetration (*P* = 0.001) (Figure 5C). Similarly, attraction and penetration of dsRNA soaked worms into tomato roots in seedling trays resulted in the significant reduction of nematode penetration after 72 h of inoculation. The findings demonstrated that the silencing of fusion gene significantly reduced the nematode attraction and penetration more than other treatments (*P* = 0.001) (Supplementary Figure S1).

### Effect of *in vitro* Gene Silencing on *M. incognita* Infecting Adzuki Bean

**FIGURE 6:**
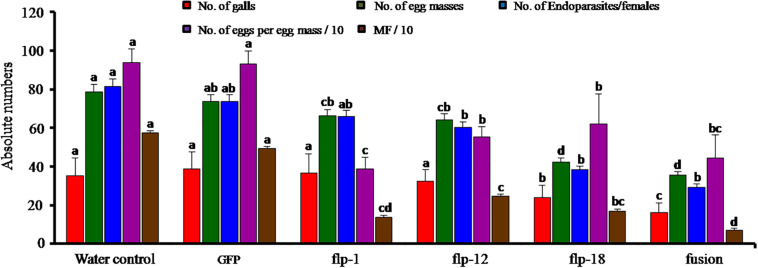
Effect of *in vitro* silencing of FLPs gene and their fusion gene on developmental and reproduction of *M. incognita* in adzuki bean. Total numbers of galls, total endoparasites, egg masses, eggs per egg mass and the respective multiplication factor (MF) of *M. incognita* in adzuki bean infected with dsRNA-treated J2s at 35 DPI. Values are means ± standard errors (*n* = 9) and letters for a given variable differed significantly amongst treatments at *P* < 0.05. J2s treated with GFP dsRNA and worms in water are used as controls.

Root-knot nematode bioassay was carried out on adzuki beans in the CYG seed growth pouch system. After 35 DPI, each plant was scored for disease parameters in terms of total number of galls, endoparasites/females, egg masses and eggs per egg mass. Target gene silencing through soaking in dsRNA of three FLP genes singly and fusion gene resulted in reduction in average galling by 54% compared to the control. This also led to reduction in total endoparasites/females and egg masses by 63% and 54%, respectively (*P* = 0.01). A significant difference was observed between the fusion dsRNA soaked J2s compared to all other treatments (*P* = 0.01). However, within single gene silenced nematodes, *Mi-flp18* was significantly different from *Mi-flp1* and *Mi-flp12* (*P* = 0.01). All the treatments demonstrated considerable differences from each other and showed reduction in nematode reproduction up to 58% (*P* = 0.01) (Figure 6).

### Host-Delivered RNAi of Fusion Gene Having Three FLPs *viz., Mi-flp1, Mi-flp12*, and *Mi-flp18*

Based on the outcome of *in-vitro* RNAi experiments, *in-planta* functional validation analyses have been carried out with FLPs-fusion gene. A commercially synthesized fusion gene using coding nucleotide sequences of *Mi-flp1*, *Mi-flp12*, and *Mi-flp18* was cloned and confirmed by sequencing. FLPs-fusion gene cassette cloned into RNAi Gateway vector pB7GWIWG2(II) is shown in Supplementary Figure S2A. PCR with different sets of primers confirmed the orientation of the sense and antisense strands of the fusion gene in the hpRNA construct.

Tobacco cv. Petit Havana was transformed with hpRNA construct harboring the fusion gene using a standardized leaf-based regeneration and transformation method and T_0_ plants were generated (Supplementary Figure S2B). To confirm the complete T-DNA integration into the T_0_ plants, initial analysis was carried out using PCR analyses with different sets of primers. Presence of the fusion gene, promoter, terminator and the selectable marker gene was determined in all the independent events and no amplification was detected in WT plant (Supplementary Figure S3).

The transgenic T_1_ plants were identified and set up by selfing the positive T_0_ plants and re-confirmed via PCR with the same set of primers as stated above, which amplifies the expected target fragments in the tested events, signifying integration of the fusion gene construct in the progeny plants (Supplementary Figure S4).

**FIGURE 7:**
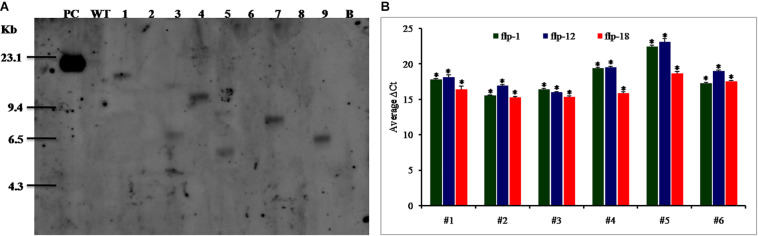
**(A)** Southern blot analysis of transgenic lines harboring dsRNA construct of FLPs-fusion gene. Lanes 1–9 shows transgenic lines 25-6, 26-2, 27-4, 28-9, 36-12, 37-1, 38-5, 40-5, and 47-2. PC – positive control (linearized recombinant vector pB7GWIWG2(II) (25 pg); WT – negative control (wild-type plant); B – blank, **(B)** Expression analysis of target genes in Southern positive T_1_ plants. *Mi-flp1, Mi-flp12*, and *Mi-flp18* expressing lines (**#1** to **#6** represent lines– 25-6, 27-4, 28-9, 36-12, 38-5, and 47-2). ΔCt values were calculated using difference in the Ct mean of target gene and reference gene (tobacco *18S rRNA*). Each bar represents the mean ± SE of *n* = 3, and asterisks shows significant difference at *P* < 0.05. Higher ΔCt values specify the lower expression of transgene in the corresponding events.

In order to confirm the pattern of transgene integration and copy number in T_1_ plants, PCR positive plants were validated by Southern blot analysis. It was observed that the transgenic lines, *viz.*, 25–6, 28–9, 38–5, and 47–2 showed single copy insertions while 27–4 and 36–12 exhibited double copies of the transgene. There was absence of any hybridization in the WT plants (Figure 7A).

Further, transgenic plants expressing fusion gene construct were confirmed by qRT-PCR. The results indicated significant expression of a transgene in all the selected events, and among them events 27–4 and 28–9 had the highest expression level with all three tested genes and the event 38–5 showed the least in terms of average ΔCT values (Figure 7B). There was no transcript detected in WT plants.

### Bioefficacy Analysis of T_1_ Transgenics of *Nicotiana tabacum* Against *M. incognita*

**FIGURE 8:**
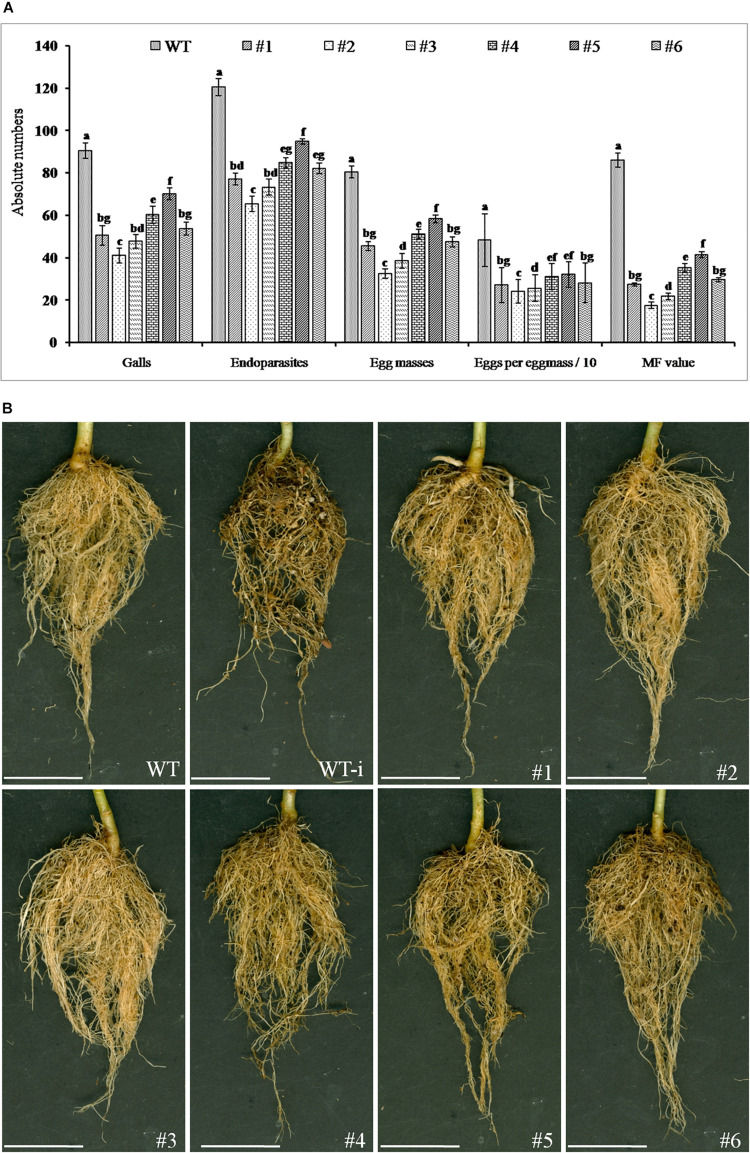
Effect of host-delivered RNAi of FLPs-fusion gene on development and reproduction of *M. incognita*. **(A)** Performance of transgenic plants was assessed in terms of number of galls, total endoparasites, egg masses, eggs per egg mass, and multiplication factor (MF) in different transgenic lines (**#1**- 25-6, **#2**- 27-4, **#3**- 28-9, **#4**- 36-12, **#5**- 38-5, and **#6**- 47-2) and WT plants at 35 DPI in soil. Each bar represents the mean ± SE of *n* = 5, and bars with different letters (within each parameter) indicate significant difference at *P* < 0.05. **(B)** Comparison of *M. incognita* infection in roots of transgenic and WT plants at 35 DPI; representative roots of (WT) healthy wild-type plants, (WT-i) nematode infected wild-type plants, transgenic events **#1 to #6**: 25-6, 27-4, 28-9, 36-12, 38-5, and 47-2 infected with *M. incognita*, scale bar = 5 cm.

T_1_ generation plants of positive transgenic events were evaluated against *M. incognita.* The nematode infection was determined after 35 DPI in terms of total number of galls, endoparasites, egg masses, and eggs per egg masses. The results showed average galling in the range of 41–70 in different T_1_ lines compared to WT plants which was observed to be about 90 that led to a reduction of 22–54% in average galling in transgenic events. This eventually resulted in significant lowering in total endoparasites in the range of 65–95 in T_1_ lines compared to about 120 in WT plants. As a result, 21–46% reduction was observed in total endoparasites in the transgenic events. Similarly, the average number of egg masses and eggs per egg mass was present in the range of 28–58 and 214–320 in T_1_ plants of different transgenic events whereas WT plants showed about 80 and 482, respectively. This resulted in significant reduction in fecundity in the transgenics events by 25–65% and 32–56%, respectively. Finally, derived MF was in range of 13.3–41.3 in T_1_ lines while WT plants documented about 86.02 which demonstrated a reduction of 85% in transgenic events compared to the WT plants (Figures 8A,B).

### Expression Analysis of Transgene in the Nematode Extracted From Transgenic Plants

**FIGURE 9:**
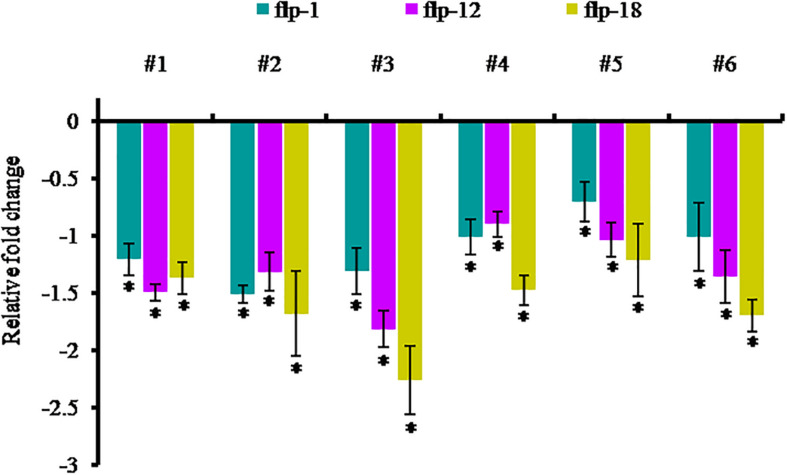
Transcript levels of *Mi-flp1, Mi-flp12*, and *Mi-flp18* in *M. incognita* females extracted from dsRNA expressing transgenic events (**#1** to **#6**: 25-6, 27-4, 28-9, 36-12, 38-5, and 47-2). Expression was measured as relative fold-change, analyzed using 2^– Δ^ ^Δ^ ^*CT*^ method. Each bar represents the mean ± SE of *n* = 3, and asterisks specify the significant difference at *P* < 0.05.

In order to examine the effect of host-delivered RNAi on suppressing the target transcripts in the worms, qRT-PCR was carried out with adult females of *M. incognita* dissect out from the transgenic plants. The expression of *Mi-flp1*, *Mi-flp12*, and *Mi-flp18* in *M. incognita* females was down-regulated significantly (*P* < 0.05) by 0.7 ± 0.2 to 1.5 ± 0.1 fold in case of *Mi-flp1*, 0.9 ± 0.1 to 1.8 ± 0.2 fold in case of *Mi-flp12*, and 1.2 ± 0.2 to 2.2 ± 0.3 fold in case of *Mi-flp18*, respectively, compared to females from WT plants (Figure 9).

## Discussion

Here, we have evaluated the role of three FLP genes of *M. incognita*, *Mi-flp1, Mi-flp12*, and *Mi-flp18* singly and in combination for their effect on nematode reproduction and parasitism. Neuropeptides, mainly FLPs, are associated with neurosensory motor function, feeding, secretion, locomotion and reproduction necessary for parasitism (Maule et al., 2002; Kimber et al., 2007; Johnston et al., 2010). Thus, FLPs interference can be directed to disruption of several functions. The conservation of FLPs genes among nematodes and its probable role in locomotion and reproduction has attracted attention for exploitation of these neuromodulator genes as possible targets for nematode management which is environmentally friendly and target specific (Piggott et al., 2011; Peymen et al., 2014; Morris et al., 2017). Quite a few neuropeptides have been identified in PPNs, and FMRFamide-like immune reactivity has also been recorded in the nervous systems of *Heterodera glycines*, *Globodera rostochiensis*, *G. pallida* and *M. incognita* and *M. graminicola* (Atkinson et al., 1988; Maule et al., 2002; McVeigh et al., 2005; Kimber et al., 2007; Dalzell et al., 2010; Holden-Dye and Walker, 2011; Atkinson L. E. et al., 2013; Papolu et al., 2013; McCoy et al., 2014; Kumari et al., 2017; Warnock et al., 2017). So far, nineteen FLPs have been identified in the *M. incognita* and out of which six have been established to have transcriptional evidence (Abad et al., 2008) and host-delivered RNAi of *Mi-flp14* and *Mi-flp18* in *N. tabacum* provided excellent reduction in nematode parasitism and reproductive potential (Papolu et al., 2013).

In this direction, present work is focused on initial functional characterization of three *flp* genes, *Mi-flp1, Mi-flp12*, and *Mi-flp18* of *M. incognita* singly and in combination employing a well established *in vitro* RNAi strategy to assess the comparative effect on nematode parasitism. Subsequently, three FLPs integrated into a fusion gene is used for silencing through host-delivered RNAi in tobacco to establish any synergistic or additive effects of the simultaneous silencing of three genes on *M. incognita*. The target dsRNA sequences were analyzed in dsCheck database to find out any off-target sites in the existing database, and no similarity was detected for the processed siRNAs. Thus, silencing of these genes possibly will not produce any off-target effects on another organisms and signifies the rational design of dsRNA molecule in turn to decrease the feasible risk.

The three FLPs transcripts were differentially expressed in different developmental stages of *M. incognita* and attain maximum expression in infective J2s, in contrast to basal level expression in eggs. Further, expression was down-regulated in J3/J4 and young females. Similarly, Papolu et al. (2013) observed higher expression of *Mi-flp14* and *Mi-flp18* in pre-parasitic J2s. These results re-emphasize the importance of FLPergic system in the initial parasitic process of *M. incognita*.

*In situ* hybridization assay in the present study revealed *Mi-flp1* expression in ventral pharyngeal nerve cord near the metacorporeal bulb of *M. incognita* J2s. Expression of *Mi-flp1* in some common nerve cells connecting the amphidial nerves to the central nervous system revealed its function similar to that of *Mi-flp18*. In *G. pallida, Gp-flp1* expression was shown to be present in different nerve cell bodies’ *viz*., PHA, PVQ, LUA, PHB, ALN, and PVC compared to *C. elegans*. Two nerves (PHA and PHB) are ciliated and innervate the phasmids in the caudal region. ALN runs laterally from the tail to the nerve ring and has central synapses with motor-neurons innervating musculature in the head of *C. elegans* (Rogers et al., 2003). Our findings are in line with earlier reports in *G. pallida* by Kimber et al. (2002). Hence, it is possible that *Mi-flp1* could have similar functional role in *M. incognita*. Previously, we had reported the expression site of *Mi-flp12* and *Mi-flp18* in the nerve cells, involved in the various biological and physiological process of *M. incognita* J2s (Banakar et al., 2015; Banakar and Rao, 2016). These findings support the involvement of the three FLPs in locomotion, host finding, reproduction and other physiological roles in the nematodes (White et al., 1986; Kimber et al., 2002, 2007; Rogers et al., 2003; Johnston et al., 2010; Kumari et al., 2017).

RNAi has been the mainstay tool for functional validation of genes in phytonematodes (Lilley et al., 2007; Dutta et al., 2015a, b). However, reports of gene silencing to explore the function of FLPergic molecules in plant nematodes are finite (Kimber et al., 2007; Dalzell et al., 2010; Atkinson N. J. et al., 2013; Papolu et al., 2013; Dong et al., 2014; Banakar et al., 2015). Here, we disrupted three individual FLPs and their combinatorial fusion gene through *in vitro* RNAi (dsRNA soaking) in *M. incognita* J2s which resulted in perturbed the expression of target transcripts of each of the FLPs. These transient natures of gene silencing effects detected in our study are in line with the earlier report of Kimber et al. (2007). Similarly, de Souza Júnior et al. (2013) reported the concurrent silencing of three proteases using a single dsRNA expression cassette that resulted in unexpected up-regulation of *Mi-asp1* during combinatorial silencing of three genes (de Souza Júnior et al., 2013). Likewise, Bakhetia et al. (2008) found an increase in the dg13 expression during the combinatorial RNAi evaluation of dg13 and dg14 genes of *H. glycines*. The possible reason underlying an expected upregulated expression during both single and combinatorial silencing of three *flps* in the present work could be due to interaction between FLPs in different biological process. *In-silico* analysis using string database revealed that *flp1, flp12*, and *flp18* are co-expressed in *C. elegans*, and *flp18* together with *flp1* plays a homeostatic role by acting on the GABAergic neural transmission at neuromuscular junctions to inhibit over excitation of the locomotor circuit. Further, we have demonstrated the successful processing of combinatorial dsRNA by northern blot analysis in the fusion dsRNA soaked worms that showed the presence of siRNA of each of the three *flps* in *M. incognita* J2s. This is the first established report in PPNs showing positive gene silencing evidence by northern blot hybridization and combinatorial dsRNA processing into siRNA.

The effect of *in vitro* silencing of *Mi-flp1*, *Mi-flp12*, *Mi-flp18* and the fusion genes on attraction and penetration of J2s approaching tomato roots was examined on PF-127 and also in soil. Worms treated with dsRNA corresponding to both individual genes and fusion cassette markedly defer in level of attraction and penetration of J2s indicating that the target genes are important in early plant-nematode interactions. Fusion gene silencing is the most effective in terms of reduction of nematode attraction and penetration compared to single gene silenced and GFP dsRNA treated controls. This establishes the synergistic effect due to simultaneous knockdown of three FLPs particularly on chemo-sensation and host finding ability of the nematode. Similar phenotypic effects were observed due to RNAi silencing of *gp-flp1, gp-flp12*, and *gp-flp18* in *G. pallida* that completely inhibited the migratory behavior after 24 h of incubation (Kimber et al., 2007). The significance of *Mi-flp18* in *M. incognita* host finding (chemotaxis), migration and infection was also established through *in vitro* RNAi previously from our lab by Papolu et al. (2013), and Banakar et al. (2015) and the results are similar to the present finding. In *C. elegans*, analysis of *flp18* and the related nerve cell mutants were defective in chemo-sensation, dauer formation, foraging and fat accumulation and also showed reduced consumption of oxygen (Albertson and Thompson, 1976; Avery and Horvitz, 1990; Avery, 1993; Tsalik and Hobert, 2003; Wakabayashi et al., 2004; Gray et al., 2005; Cohen et al., 2009). Here, the observed decrease in nematode migration and penetration could be attributed to the gene silencing effect of FLPs that interrupted neuromotor actions involved in olfaction, chemotaxis and infectivity.

The reduced infection after knockdown of individual FLPs and their fusion gene also resulted in considerable retardation of infection, development and multiplication of *M. incognita* in adzuki bean. Amongst treatments, the fusion gene was the most effective in reducing the infection as indicated by least number of total endoparasites in the roots and significantly affected the fecundity in reduction of eggs per egg mass. The result was comparable to the penetration assays on tomato roots. Our studies could be supported by Moffett et al. (2003) in *C. elegans* where *flp1* inhibits rhythmic contractions of the ovijector, the organ that controls egg-laying when *flp1* was tested in different concentrations on ovijector. These reports strongly suggest that *Mi-flp1, Mi-flp12*, and *Mi-flp18* are involved in nematode perception of host plants, feeding and reproduction. Therefore, all three FLPs could be potential targets for future management of *M. incognita*.

On confirming the *in vitro* RNAi silencing of *Mi-flp1, Mi-flp12*, *Mi-flp18* and fusion gene, additional proof for the possibility of simultaneous silencing of three genes and consequent effects on *M. incognita* was undertaken by *in planta* validation with a highly effective tobacco system. Using *Agrobacterium*-mediated genetic transformation, transgenic plants were generated expressing FLPs-fusion dsRNA. Molecular characterization confirmed the presence, integration and inheritance of the T-DNA harboring the hpRNA construct independently in six transformed lines. Though, the transcripts abundance at mRNA level of transgene measured by qRT-PCR was variable amongst events, signifying that the transgene was integrated at random sites in the plant genome. Additionally, resistance offered due to the expression of transgene was confirmed by bioefficacy analysis. Bioefficacy analysis indicated a pronounced decrease in *M. incognita* development and reproduction in most of the T_1_ lines compared to WT plants with up to 85% reductions in derived MF. The significant reduction of MF in transgenic lines has proved efficient RNAi silencing of FLPs-fusion gene. Earlier study by Papolu et al. (2013) demonstrated that tobacco plants expressing RNAi construct of the single gene *Mi-flp18* had reduced the numbers of galls and final MF compared to WT plants. The present findings are better than the earlier reports in reducing the MF in transgenic lines and on par with other parameters observed in *M. incognita*. In the present study, silencing of more than one gene at a time with multiple roles had a higher impact on reducing the nematode parasitism in plants. The pyramiding expression of three FLP genes involved in more than one function produced synergistic effects by a detrimental effect on development of *M. incognita*. Our results are supported by the finding of Urwin et al. (1998), where transgenic *Arabidopsis* expressing a stacked gene construct having a cowpea trypsin inhibitor and a cystatin displayed improved resistance to *H. schachtii*. Similarly, a dsFusion RNAi construct having three proteases gene *viz., Mi-ser1*, *Mi-cpl1*, and *Mi-asp1* interfere the nematode fecundity and successfully reduced disease burden in tobacco. These studies demonstrated the gene pyramiding in transgenic plants expressing more than one gene not only offer two defense systems across worms but also widen the spectrum of resistance to different nematode species (de Souza Júnior et al., 2013). Transgenic plants were morphologically similar and comparable to WT-plants, indicating the specificity of gene silencing.

In order to assess the host delivered silencing effect of *Mi-flp1, Mi-flp12*, and *Mi-flp18* on nematode development, adult females were dissect out from T_1_ transgenic plants and WT-plants and subjected to qRT-PCR analysis. A considerable reduction (up to 2.2-fold) in gene expression was observed in the young females from transgenic plants compared to that from WT-plants. The current findings are is in confirmation with the previous reports showing inhibitory effect of *flp1* and other 15 *flp* genes in modulating ovijector of *A. suum* by cessation of contractile activity of oviduct, affecting egg laying (Moffett et al., 2003). Loss of *flp1* gene in *C. elegans* resulted in reduced locomotion and egg laying rate (Chang et al., 2015) that supports the findings of present work.

Although, there are some reports showing efficacy of FLPs for disrupting the neuromotor functions, to the best of our knowledge, this is the first report to demonstrate effectiveness of *Mi-flp1* and *Mi-flp12* and their fusion gene along with *Mi-flp18* for the management of *M. incognita* using *in vitro* gene silencing as well as host-induced gene silencing. Significant reduction in nematode MF, due to knockdown of FLPs would be of enormous value to bring down the resident population pressure in soil for the successive crops. Given the transient nature of RNAi effects, it is practically not possible to attain absolute nematode resistance (Rosso et al., 2009). As RKNs complete 3–4 or more generations during a cropping season, about 60% decline in multiplication is adequate to reduce nematode population below the economic threshold (Fuller et al., 2008). Additionally, sustainable management can be accomplished by pyramiding few *flp* genes for efficient interference of various physiological processes requisite for successful completion of nematode lifecycle.

## Data Availability Statement

The raw data supporting the conclusions of this article will be made available by the authors, without undue reservation.

## Author Contributions

UR conceived, designed, and supervised the experiments. PB and AH performed all the experiments. PP carried out Northern blot analysis. AH analyzed the data and wrote the original draft of the manuscript. UR, AH, and PB finalized the draft of the manuscript. All authors read and approved the final manuscript.

## Conflict of Interest

The authors declare that the research was conducted in the absence of any commercial or financial relationships that could be construed as a potential conflict of interest.

## Footnotes

1 https://mega-international.com/tech-info/

## Supplementary Material

Supplementary Figure 1 Effect of *in vitro* RNAi on host invasion of *M. incognita* in soil. Penetration of dsRNA soaked worms into roots of tomato seedlings after 72 h of inoculation. Each bar represents the mean standard error (*n* = 6); bars with letters indicate significant difference at *P* > 0.05. Nematodes treated with GFP dsRNA (non-native control) and worms in water were used as controls.

Supplementary Figure 2 **(A)** T-DNA region of binary vector used for FLPs-fusion gene silencing in tobacco cv. Petit Havana. RNAi suppression construct – pB7GWIWG2(II). Inverted repeats of fusion gene fragment were cloned into the destination vector. RB – T-DNA right border, LB – left border, *bar* – selectable marker gene, P35S – Cauliflower mosaic virus (CaMV) 35S promoter, T35S – CaMV 35S terminator, fusion gene – *M. incognita* FMRFamide like peptides (FLPs) fusion gene, attB1 and attB2 – LR reaction sites, **(B)** *Agrobacterium*- mediated transformation and regeneration of tobacco cv. Petit Havana. **(a)** Leaf explants of 1 cm^2^ cut from young tobacco leaves **(b)** callusing of co-cultivated explants **(c)** selection of transformed calli in presence of glufosinate **(d)** regeneration of selected explants **(e)** appearance of roots in rooting medium **(f)** hardened plants in glasshouse **(g)** successful plant growth and maturation **(h)** fruiting and seed setting.

Supplementary Figure 3 PCR analysis of genomic DNA extracted from the primary transgenic plants harboring RNAi–fusion gene cassette. **(A)** Amplification of the target gene using gene specific primers, **(B)** Amplification of sense strand using primers 35S promoter forward and *attB2* reverse, **(C)** Amplification of the antisense strand using primers 35S terminator forward and *attB2* reverse, **(D)** Amplification of *bar* gene to confirm the presence and inheritance of dsRNA into transformed plants. M – 100 bp molecular marker. WT – negative control (WT-plant); Lanes 1–13- independent T_0_ events (25, 26, 27, 28, 36, 37, 38, 40, 47, 48, 52, 53, and 84).

Supplementary Figure 4 PCR analysis of genomic DNA extracted from the T_1_ transgenic lines harboring RNAi–fusion gene cassette. M – 100 bp molecular marker; WT – negative control (WT-plant); Lanes 1–12 – show independent progenies of T_0_ events (25-6, 26-2, 27-4, 28-9, 36-12, 37-1, 38-5, 40-5, 47-2, 48-3, 52-7, and 53-1). **(A)** Target gene amplification, **(B)** *bar* gene amplification.
