# Supplementary material for: Simultaneous RNAi Knockdown of Three FMRFamide-Like Peptide Genes, Mi-flp1, Mi-flp12, and Mi-flp18 Provides Resistance to Root-Knot Nematode, Meloidogyne incognita
Source: Front Microbiol. 2020 Oct 23;11:573916. doi: 10.3389/fmicb.2020.573916 (PMC7644837; doi:10.3389/fmicb.2020.573916)
Supplement: Supplementary Figure 1 — Effect of in vitro RNAi on host invasion of M. incognita in soil. Penetration of dsRNA soaked worms into roots of tomato seedlings after 72 h of inoculation. Each bar represents the mean standard error (n = 6); bars with letters indicate significant difference at P > 0.05. Nematodes treated with GFP dsRNA (non-native control) and worms in water were used as controls. [file Data_Sheet_1.docx]

**Supplementary Data sheet 1**

**Figure S1 Effect of *in vitro* RNAi on host invasion of *M. incognita* in soil.** Penetration of dsRNA soaked worms into roots of tomato seedlings after 72 h of inoculation. Each bar represents the mean standard error (n=6); bars with letters indicate significant difference at P ˃ 0.05. Nematodes treated with GFP dsRNA (non-native control) and worms in water were used as controls.

**Figure S2 (A) T-DNA region of binary vector used for FLPs-fusion gene silencing in tobacco cv. Petit Havana**. RNAi suppression construct - pB7GWIWG2(II). Inverted repeats of fusion gene fragment were cloned into the destination vector. RB – T-DNA right border, LB – left border, *bar* – selectable marker gene, P35S – Cauliflower mosaic virus (CaMV) 35S promoter, T35S – CaMV 35S terminator, fusion gene – *M. incognita* FMRFamide like peptides (FLPs) fusion gene, attB1 and attB2 – LR reaction sites, **(B) *Agrobacterium*- mediated transformation and regeneration of tobacco cv. Petit Havana. (a)** Leaf explants of 1 cm^2^ cut from young tobacco leaves **(b)** callusing of co-cultivated explants **(c)** selection of transformed calli in presence of glufosinate **(d)** regeneration of selected explants **(e)** appearance of roots in rooting medium **(f)** hardened plants in glasshouse **(g)** successful plant growth and maturation **(h)** fruiting and seed setting.

**Figure S3 PCR analysis of genomic DNA extracted from the primary transgenic plants harboring RNAi–fusion gene cassette. (A)** Amplification of the target gene using gene specific primers, (**B)** Amplification of sense strand using primers 35S promoter forward and *attB2* reverse, (**C)** Amplification of the antisense strand using primers 35S terminator forward and *attB2* reverse, (**D)** Amplification of  *bar* gene to confirm the presence and inheritance of dsRNA into transformed plants. M – 100 bp molecular marker. WT – negative control (WT-plant); Lanes 1 to 13- independent T_0_ events (25, 26, 27, 28, 36, 37, 38, 40, 47, 48, 52, 53, 84).

**Figure S4 PCR analysis of genomic DNA extracted from the T_1_ transgenic lines harboring RNAi–fusion gene cassette.** M – 100 bp molecular marker; WT – negative control (WT-plant); Lanes 1 to 12 – show independent progenies of T_0_ events (25-6, 26-2, 27-4, 28-9, 36-12, 37-1, 38-5, 40-5, 47-2, 48-3, 52-7, 53-1). **(A)** Target gene amplification, **(B)** *bar* gene amplification.


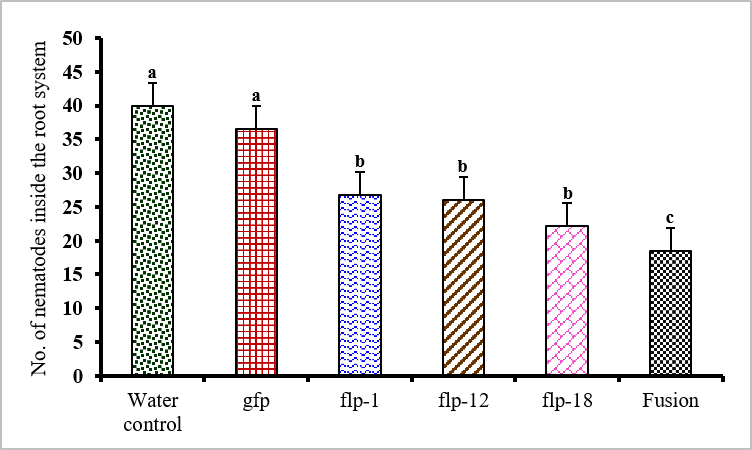


**FIGURE S1**


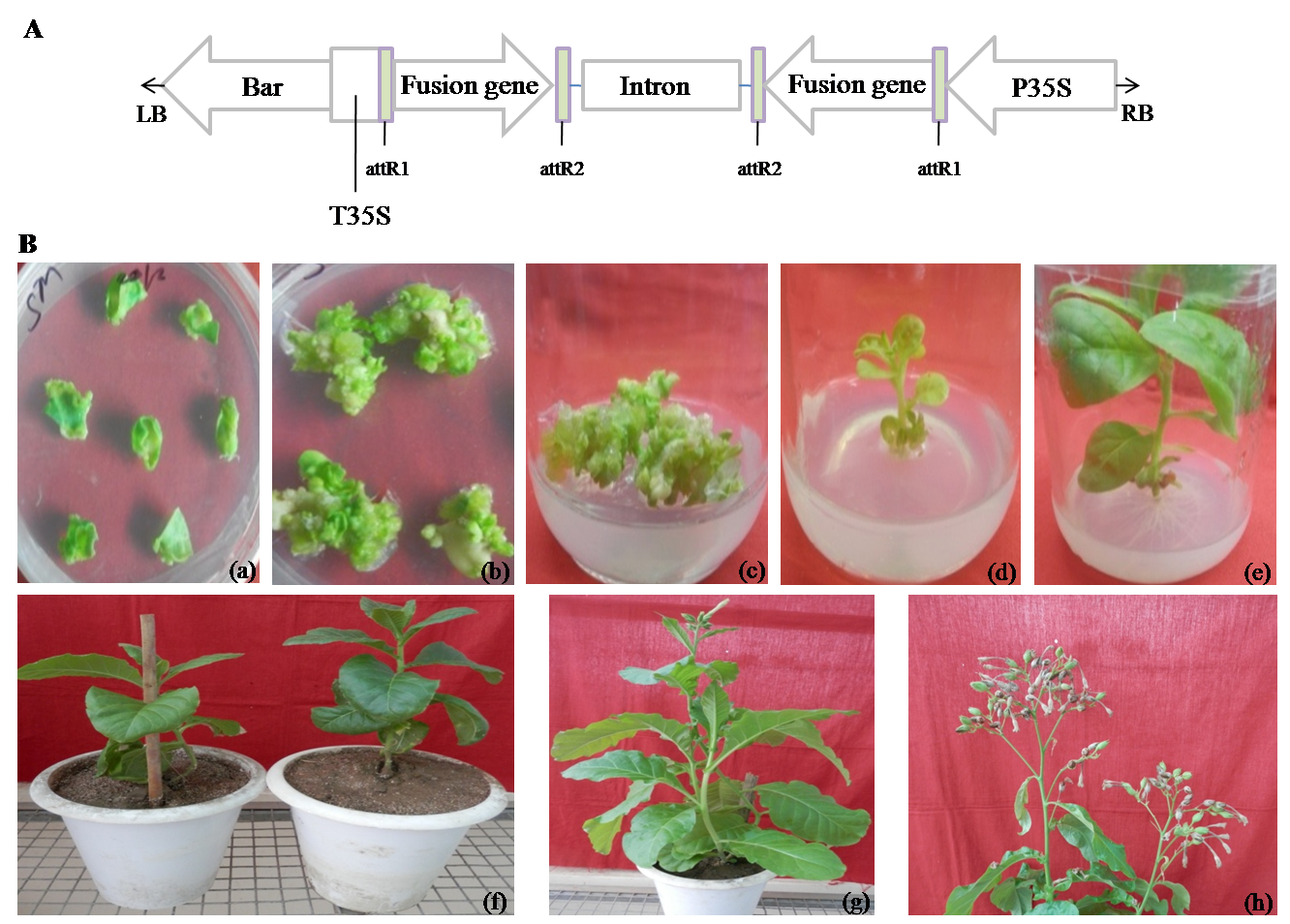


**FIGURE S2**


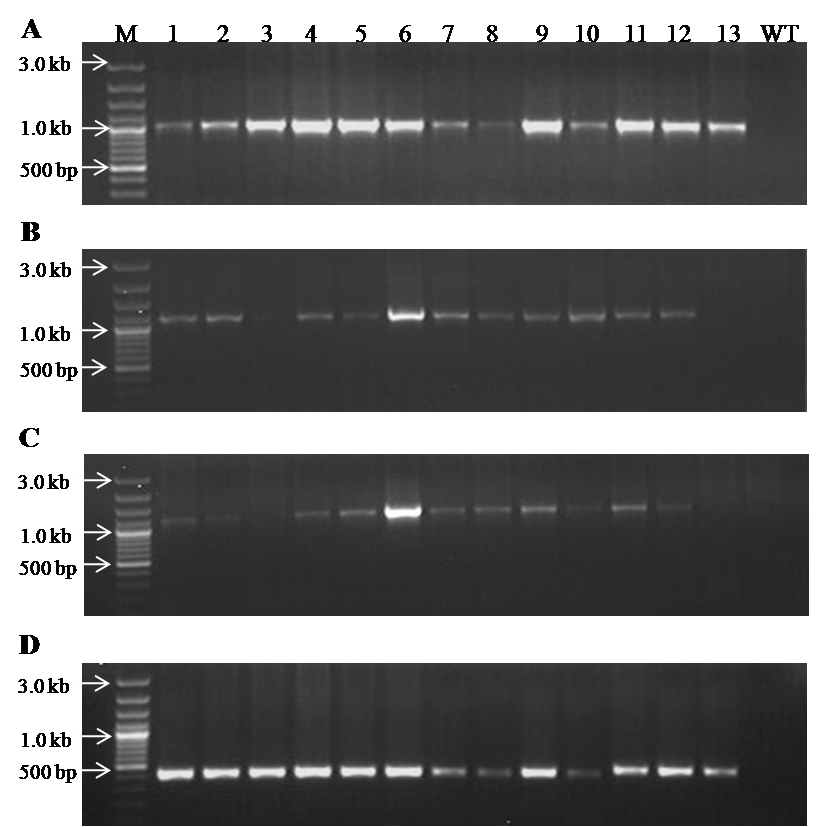


**FIGURE S3**


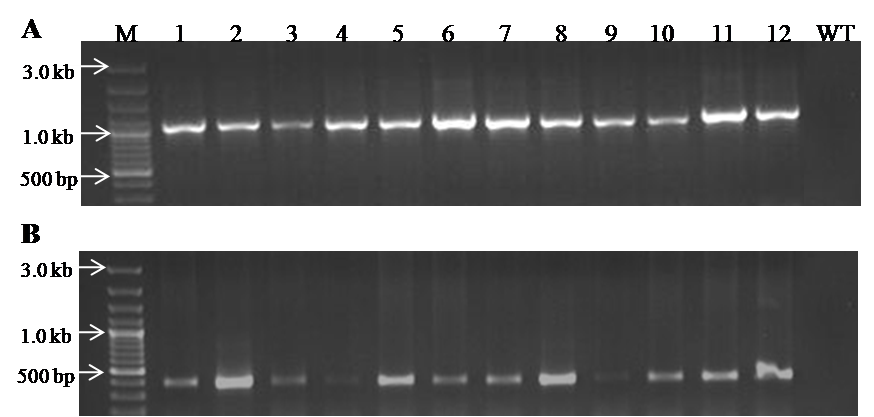


**FIGURE S4**

**Table 1** List of primers used for PCR and qRT-PCR

| **Sl. No** | **Gene name** | **Primers sequences** | **Tm** | **Product size** |
| --- | --- | --- | --- | --- |
| 1 | M-13 | Forward : 5′- GTTTTCCCAGTCACGAC -3′ | 55 | - |
|  |  | Reverse : 5′- TCACACAGGAAACAGCTATGAC -3′ |  |  |
| 2 | GFP | Forward : 5′- AGCGGCACGACTTCTTCA -3′ | 60 | 375 |
|  |  | Reverse : 5′- GTGTGGACAGGTAATGGTTGT -3′ |  |  |
| 3 | GFP-qRT | Forward : 5′- GAAACATCCTCGGCCACAAG -3′ | 60 | 185 |
|  |  | Reverse : 5′- TAAAAGGACAGGGCCATCGC -3′ |  |  |
| 3 | *18S rRNA*-*Mi*-qRT | Forward : 5′- TCAACGTGCTTGTCCTACCCTGAA -3′ | 60 | 155 |
|  |  | Reverse : 5′- TGTGTACAAAGGGCAGGGACGTAA -3′ |  |  |
| 4 | *18S rRNA*-  Tobacco-qRT | Forward : 5’- CGCGCGCTACACTGATGTATTCAA -3’ | 60 | 172 |
|  |  | Reverse : 5’- TACAAAGGGCAGGGACGTAGTCAA -3’ |  |  |
| 5 | *Mi-flp1* | Forward : 5′- ATTCCTGGTCAGATGCAGACCCAA -3′ | 60 | 232 |
|  |  | Reverse : 5′- CCACTACTTCGGCCAAATCGAAGA -3′ |  |  |
| 6 | *Mi-flp12* | Forward : 5′- CCCAAGTTTGAGCTCTAAAAACAC -3′ | 60 | 349 |
|  |  | Reverse : 5′- TCATCGTCCAAATCGAATGA -3′ |  |  |
| 7 | *Mi-flp18* | Forward : 5′- CGATGAAAGACCAAAACGTG -3′ | 60 | 407 |
|  |  | Reverse : 5′- ACGATGATGGAAAGGAATGG -3′ |  |  |
| 8 | *Mi-flp1*-qRT | Forward : 5′- AGATCTGCTGCCTCAAACGA -3′ | 60 | 165 |
|  |  | Reverse : 5′- ACTTTGGCCGACTAATCCCT -3′ |  |  |
| 9 | *Mi-flp12*-qRT | Forward : 5′- TTGTATCCCTAACACTTGCCAT -3′ | 60 | 208 |
|  |  | Reverse : 5′- GCGGCTCGATAATTTTGAAGC -3′ |  |  |
| 10 | *Mi-flp18*-qRT | Forward : 5′- GAATGCCTGGGGTACTTCGA -3′ | 60 | 160 |
|  |  | Reverse : 5′- CGGAGGACTTGAGGCATTCT -3′ |  |  |
| 11 | *Mi-flp1*-ISH | Forward : 5′- ATTCCTGGTCAGATGCAGACCCAA -3′ | 60 | 232 |
|  |  | Reverse : 5′- CCACTACTTCGGCCAAATCGAAGA -3′ |  |  |
| 12 | FLPs-fusion cassette | Forward : 5′- ATTCCTGGTCAGATGCAGACCCAA -3′ | 60 | 988 |
|  |  | Reverse : 5′- ACGATGATGGAAAGGAATGG -3′ |  |  |
